# IFNγ induces Bcl3 expression by JAK1/STAT1/p65 signaling, resulting in increased IL‐8 expression in ovarian cancer cells

**DOI:** 10.1002/2211-5463.13624

**Published:** 2023-07-18

**Authors:** Bijaya Gaire, Sveta Padmanabhan, Yue Zou, Mohammad M. Uddin, Suprataptha U. Reddy, Ivana Vancurova

**Affiliations:** ^1^ Department of Biological Sciences St. John's University New York NY USA

**Keywords:** Bcl3, IFNγ, interleukin‐8, JAK1, ovarian cancer, STAT1

## Abstract

We have recently shown that IFNγ, produced during cancer therapy, induces expression of the Bcl3 proto‐oncogene in ovarian cancer (OC) cells, resulting in their increased proliferation, migration, and invasion, but the mechanisms are unknown. Here, we demonstrate that the IFNγ‐induced Bcl3 expression is dependent on JAK1 and STAT1 signaling, and on p65 NFκB. Furthermore, the IFNγ‐induced Bcl3 expression is associated with an increased occupancy of Ser‐727 phosphorylated STAT1 and acetylated histone H3 at the *Bcl3* promoter. Our data indicate that Bcl3 promotes expression of the pro‐inflammatory chemokine interleukin‐8 (IL‐8) in OC cells. These findings identify Bcl3 as a novel target of IFNγ/JAK1/STAT1 signaling and suggest that targeting the JAK1/STAT1 pathway may suppress IFNγ‐induced Bcl3 expression in OC.

## Graphical abstract

Our results show that IFNγ induces Bcl3 proto‐oncogene expression, which is dependent on JAK1, STAT1, and p65 NFκB signaling, and associated with increased promoter occupancy by phosphorylated STAT1 and acetylated histone H3. The IFNγ‐induced Bcl3 promotes expression of interleukin‐8 (IL‐8) in ovarian cancer cells. These findings identify Bcl3 as a novel target of IFNγ/JAK1/STAT1 signaling and inducer of IL‐8 expression.

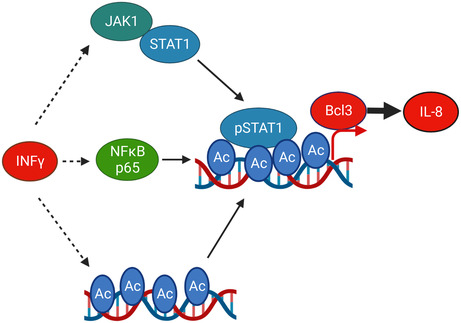

**Abbreviations**
ChIP: chromatin immunoprecipitation
ICB: immune checkpoint blockade
IFNγ: interferon‐γ
IL‐8: interleukin‐8
ISGs: IFNγ‐stimulated genes
OC: ovarian cancer
qRT‐PCR: quantitative real‐time RT‐PCR
Rux: ruxolitinib
TSS: transcription start site
UT: untreated
WCE: whole‐cell extract

The proto‐oncogene Bcl3 is an atypical member of IκB family, which, unlike other IκB proteins, localizes predominantly in the nucleus [1, 2]. Bcl3 can be recruited to gene promoters, resulting in transcriptional activation or repression, depending on composition of the transcriptional complexes [3, 4, 5, 6, 7]. Genes regulated by Bcl3 include genes involved in regulation of cell survival, proliferation, and immune escape [8, 9]. The Bcl3 expression is increased in hematological malignancies [10, 11, 12, 13, 14] and many types of solid tumors including ovarian cancer (OC) [15, 16, 17, 18, 19, 20, 21]. Since the increased expression of Bcl3 in cancer cells promotes their proliferation, migration, invasion, and metastases, Bcl3 has become an important target for development of anticancer strategies [9, 22].

As other members of IκB family, the BCL‐3 transcription is regulated by NFκB signaling and upregulated in response to stimulation with TNFα, interleukin (IL)‐1, IL‐6, and other pro‐inflammatory cytokines [10, 15, 23, 24, 25, 26, 27]. We have recently shown that the Bcl3 expression is increased in OC cells and tissues, and promotes expression of the immune checkpoint PD‐L1 [9]. In addition, the Bcl3 expression in OC cells can be further increased by interferon‐γ (IFNγ), resulting in increased survival, proliferation, and migration of OC cells [9]. However, the mechanisms of how IFNγ induces the Bcl3 expression are unknown.

Interferon‐γ is a pleiotropic cytokine that can have, depending on the cellular and molecular context, both antitumor and pro‐tumorigenic functions [28, 29, 30, 31]. Because of its antitumor potential, IFNγ has been used in cancer treatment [32, 33, 34, 35]. In addition, IFNγ expression can be induced in response to radiation therapy or immune checkpoint blockade (ICB) used in cancer treatment [36, 37, 38]. Considering the strong oncogenic potential of Bcl3, in this study, we have investigated the mechanisms by which IFNγ induces the Bcl3 expression.

Our results demonstrate that the IFNγ‐induced Bcl3 expression in OC cells is dependent on JAK1 and STAT1 signaling, as well as on p65 NFκB. In addition, our data show that the Bcl3 expression promotes expression of the pro‐angiogenic and pro‐inflammatory chemokine IL‐8/CXCL8 in OC cells. These findings identify Bcl3 as a novel target of IFNγ/JAK1/STAT1 signaling and inducer of IL‐8 expression, and suggest that targeting the JAK1/STAT1 pathway may suppress the IFNγ‐induced Bcl3 expression in OC.

## Materials and methods

### Cell culture and treatment

Ovarian cancer SKOV3 and OVCAR3 cells, obtained from the American Type Culture Collection (Rockville, MD, USA), were cultured (5 × 10^5^ cells·mL^−1^) in 6‐well plates as described [39, 40]. For treatment with IFNγ, human recombinant IFNγ (285‐IF‐100; R&D Systems, Minneapolis, MN) was reconstituted in sterile water. Cell viability was measured by trypan blue.

### Transfections with siRNA

Human JAK1 (sc‐35719), STAT1 (sc‐44123), p65 (sc‐29410), and non‐silencing control (sc‐37007) siRNAs were purchased from Santa Cruz Biotechnology (Santa Cruz, CA, USA); transfections were performed as described [40].

### Quantitative real‐time RT‐PCR

Total RNA was isolated using RNeasy Mini Kit (Qiagen, Germantown, MD, USA), and quantitative real‐time RT‐PCR (qRT‐PCR) was performed as described [40]. The primers for quantification of human *Bcl3*, *JAK1*, *STAT1*, *p65*, *IL‐8*, and *actin* mRNA were from Qiagen. The mRNA values are expressed as a percentage of control untreated (UT) samples, which were set as 100%.

### Western analysis

Whole‐cell extracts (WCEs) were prepared, and western analysis was performed as described [9, 40]. The primary antibodies were the following: JAK1 (Proteintech, Rosemont, IL, USA, #66466‐I‐Ig; dilution 1 : 500), Bcl3 (Proteintech, #23959‐I‐AP; dilution 1 : 500), STAT1 (Cell Signaling, Danvers, MA, USA, #9172; dilution 1 : 1000), Ser727 p‐STAT1 (Cell Signaling, #8826; dilution 1 : 1000), p65 NFκB (Santa Cruz, #sc‐8008; dilution 1 : 200), K314/315 ac‐p65 (Signalway Antibody, Greenbelt, MD, USA, HW136; dilution 1 : 200), histone H3 (Abcam, Cambridge, MA, USA, Ab1791; dilution 1 : 1000), Lys9 acetyl histone H3 (Cell Signaling, #9649S; dilution 1 : 1000), and control actin (Sigma, St. Louis, MO, USA, A5060; dilution 1 : 2000).

### Chromatin immunoprecipitation

Chromatin immunoprecipitation (ChIP) was performed as described [40, 41], using the following antibodies: STAT1 (Cell Signaling, #9172), Ser727 p‐STAT1 (Cell Signaling, #8826S), histone H3 (Abcam, Ab1791), Lys9 acetyl histone H3 (Cell Signaling, #9649S), p65 (Sigma, MAB3026), and K314/315 ac‐p65 (Signalway, HW136). Each immunoprecipitation was performed at least three times using different chromatin samples, and the occupancy was calculated by using human IGX1A negative control primers (SA Biosciences, Frederick, MD, USA). The results were calculated as a fold difference in STAT1, Ser727 p‐STAT1, histone H3, ac‐H3, p65, and K314/315 ac‐p65 occupancy at human *Bcl3* promoter compared with the control negative IGX1A locus that does not bind any transcription factors. The *Bcl3* primers used for real‐time PCR were as follows: F, 5‐AACTGAGAGGCAGAGAGATG‐3; R, 5‐CTGCCTCTGTTTTTGTCTTT‐3.

### ELISA assay

Human IL‐8/CXCL8 release was measured as described [40].

### Statistical analysis

The results represent at least three experiments and are presented as means ± SE. Data were analyzed by using instat software package (GraphPad, San Diego, CA, USA). Statistical significance was evaluated by using one‐way ANOVA Tukey *post hoc* test, and *P* < 0.05 was considered significant.

## Results

### IFNγ‐induced Bcl3 expression in OC cells is mediated by JAK1

We have recently shown that IFNγ induces Bcl3 expression, but the responsible mechanisms are unknown [9]. Since the main signaling pathway by which IFNγ induces gene expression is via the canonical JAK1‐STAT1 signaling pathway [28, 29, 30], we investigated whether the IFNγ induced Bcl3 expression is dependent on JAK1/STAT1 signaling. To this end, we employed two commonly used OC cell lines: the highly invasive non‐serous SKOV3 cells and the high‐grade serous OVCAR3 cells, since both cell lines exhibit high levels of Bcl3 expression, which is further increased by IFNγ [9].

**Fig. 1:**
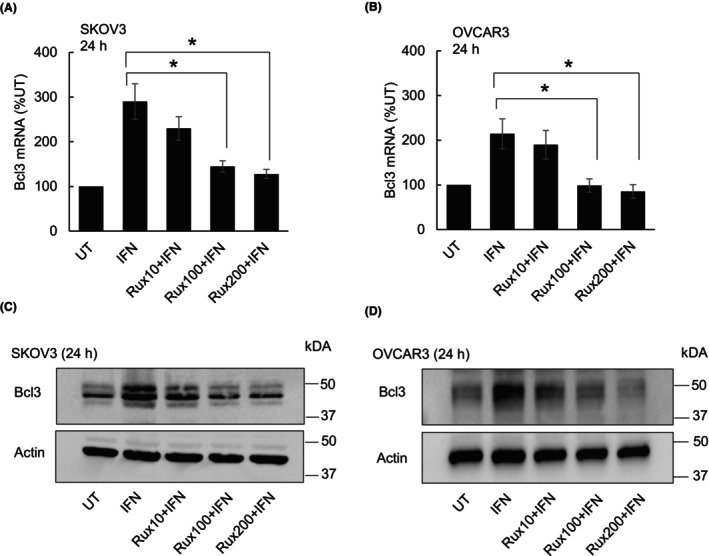
IFNγ‐induced Bcl3 expression in OC cells is dependent on JAK activity. RT‐PCR of Bcl3 mRNA levels in SKOV3 (A) and OVCAR3 (B) cells preincubated 6 h with different concentrations of JAK inhibitor, Rux (10 nm, 100 nm, and 200 nm), and treated 24 h with IFNγ (50 ng·mL^−1^). Western blotting of Bcl3 in WCE prepared from SKOV3 (C) and OVCAR3 (D) cells preincubated 6 h with different concentrations of Rux (10 nm, 100 nm, and 200 nm) and treated 24 h with IFNγ (50 ng·mL^−1^). The values in panels A and B represent the mean ± SE (*n* = 4); one‐way ANOVA Tukey *post hoc* test; an asterisk denotes a statistically significant (*P < 0.05) change.

To determine whether the IFNγ‐induced Bcl3 expression in OC cells is dependent on JAK activity, we first analyzed Bcl3 gene and protein levels in IFNγ‐treated SKOV3 and OVCAR3 cells pretreated with the JAK pharmacological inhibitor, ruxolitinib (Rux). As shown in Fig. 1, 100 nm and 200 nm Rux significantly reduced *Bcl3* mRNA levels in IFNγ‐treated SKOV3 (Fig. 1A) and OVCAR3 (Fig. 1B) cells. Furthermore, 100 and 200 nm Rux decreased the protein levels of Bcl3 in both cell types (Fig 1C,D), indicating that the IFNγ‐induced Bcl3 expression in OC cells is dependent on JAK activity.

**Fig. 2:**
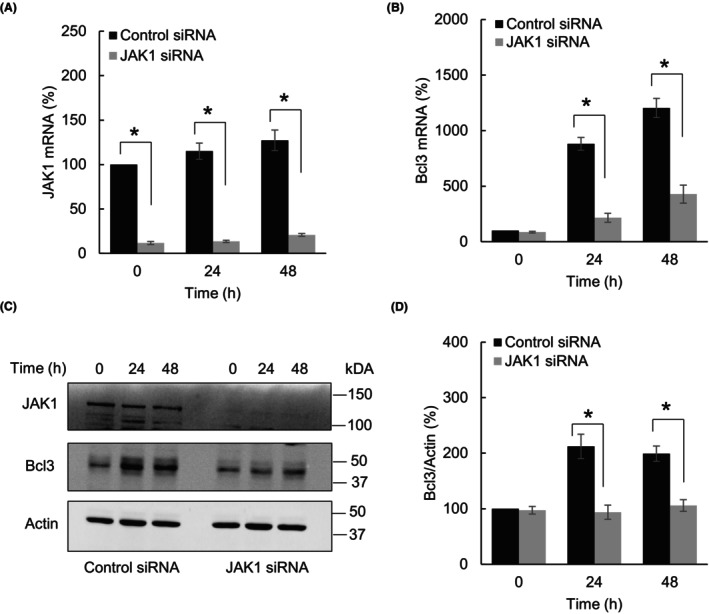
IFNγ‐induced Bcl3 expression in OC cells is mediated by JAK1. (A) JAK1 mRNA levels in SKOV3 cells transfected with control or JAK1 siRNA and treated with IFNγ (50 ng·mL^−1^). (B) Bcl3 mRNA levels in SKOV3 cells transfected with control or JAK1 siRNA and treated with IFNγ (50 ng·mL^−1^). (C) Western blotting of JAK1, Bcl3, and Actin in WCE prepared from SKOV3 cells transfected with control or JAK1 siRNA and treated with IFNγ (50 ng·mL^−1^). (D) Densitometric evaluation of Bcl3 protein levels shown in panel C. The Bcl3 densities were normalized to Actin. The values represent the mean ± SE (*n* = 3); unpaired *t*‐test; an asterisk denotes a statistically significant (*P < 0.05) change compared with cells transfected with control siRNA.

To ascertain whether the IFNγ‐induced Bcl3 expression is specifically dependent on JAK1, we analyzed the Bcl3 levels in SKOV3 cells transfected with control and JAK1‐specific siRNAs. As shown in Fig. 2, JAK1 suppression by siRNA significantly reduced both mRNA (Fig. 2A) and protein (Fig. 2C) levels of JAK1. Importantly, JAK1 suppression significantly attenuated the IFNγ‐induced Bcl3 mRNA (Fig. 2B) and protein (Fig 2C,D) levels in SKOV3 cells, indicating that the IFNγ‐induced Bcl3 expression in OC cells is mediated by JAK1.

### IFNγ‐induced Bcl3 expression in OC cells is mediated by STAT1

**Fig. 3:**
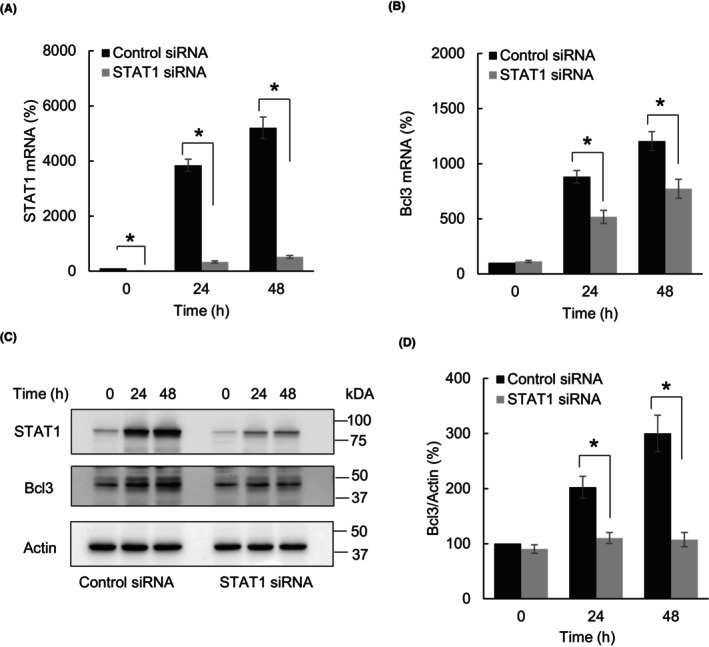
IFNγ‐induced Bcl3 expression in OC cells is mediated by STAT1. (A) STAT1 mRNA levels in SKOV3 cells transfected with control or STAT1 siRNA and treated with IFNγ (50 ng·mL^−1^). (B) Bcl3 mRNA levels in SKOV3 cells transfected with control or STAT1 siRNA and treated with IFNγ (50 ng·mL^−1^). (C) Western blotting of STAT1, Bcl3, and Actin in WCE prepared from SKOV3 cells transfected with control or STAT1 siRNA and treated with IFNγ (50 ng·mL^−1^). (D) Densitometric evaluation of Bcl3 protein levels shown in panel C. The Bcl3 densities were normalized to Actin. The values represent the mean ± SE (*n* = 3); unpaired *t*‐test; an asterisk denotes a statistically significant (*P < 0.05) change compared with cells transfected with control siRNA.

In canonical IFNγ signaling, activation of JAK1 leads to phosphorylation and activation of STAT1, which then translocates to the nucleus and induces transcription of IFNγ‐stimulated genes (ISGs) [28, 29, 30]. To determine whether the IFNγ‐induced Bcl3 expression is dependent on STAT1, we analyzed Bcl3 levels in SKOV3 cells transfected with control and STAT1‐specific siRNAs. As shown in Figs 3A,C, IFNγ increased mRNA and total protein levels of STAT1 in SKOV3 cells, and the IFNγ‐induced STAT1 expression was suppressed in STAT1 siRNA transfected cells. Importantly, suppression of STAT1 significantly reduced the IFNγ‐induced Bcl3 mRNA (Fig. 3B) and total protein (Figs 3C,D) levels, indicating that the IFNγ‐induced Bcl3 expression in OC cells is mediated by STAT1.

### IFNγ‐induced Bcl3 expression in OC cells is mediated by p65 NFκB

**Fig. 4:**
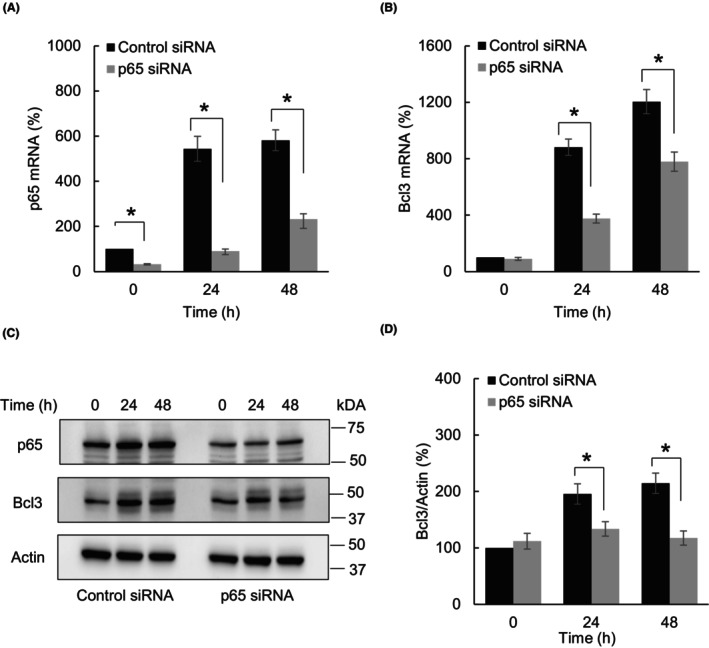
IFNγ‐induced Bcl3 expression in OC cells is mediated by p65 NFκB. (A) p65 mRNA levels in SKOV3 cells transfected with control or p65 siRNA and treated with IFNγ (50 ng·mL^−1^). (B) Bcl3 mRNA levels in SKOV3 cells transfected with control or p65 siRNA and treated with IFNγ (50 ng·mL^−1^). (C) Western blotting of p65, Bcl3, and Actin in WCE prepared from SKOV3 cells transfected with control or p65 siRNA and treated with IFNγ (50 ng·mL^−1^). (D) Densitometric evaluation of Bcl3 protein levels shown in panel C. The Bcl3 densities were normalized to Actin. The values represent the mean ± SE (*n* = 3); unpaired *t*‐test; an asterisk denotes a statistically significant (*P < 0.05) change compared with cells transfected with control siRNA.

Even though the JAK/STAT pathway is the main mechanism responsible for the expression of ISGs [28, 29, 30], several studies have shown that the IFNγ‐induced expression of NFκB‐dependent genes can be regulated also by p65 NFκB [42, 43]. To examine the possibility that the IFNγ‐induced Bcl3 expression in OC cells might be dependent on p65 NFκB, we analyzed the Bcl3 expression in IFNγ‐treated SKOV3 cells transfected with p65 siRNA. As shown in Fig. 4, IFNγ increased mRNA (Fig. 4A) and protein (Fig. 4C) levels of p65 NFκB in SKOV3 cells, and the IFNγ‐increased p65 expression was suppressed in cells transfected with p65 specific siRNA (Figs. 4A,C). Importantly, suppression of p65 significantly reduced both mRNA (Fig. 4B) and protein (Figs 4C,D) levels of Bcl3 in SKOV3 cells treated with IFNγ. These results indicate that the IFNγ‐induced Bcl3 expression in OC cells is also mediated by p65 NFκB.

### IFNγ increases Bcl3 promoter occupancy by Ser727‐pSTAT1 and ac‐histone H3

**Fig. 5:**
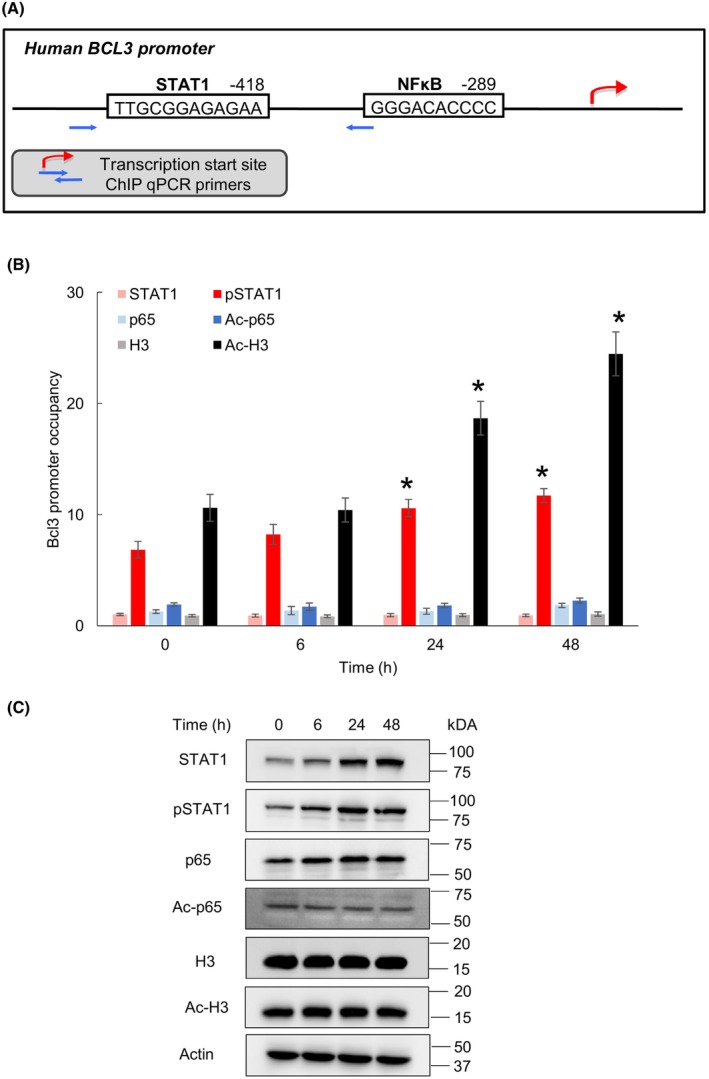
IFNγ increases *Bcl3* promoter occupancy by S727‐pSTAT1 and K9‐ac‐histone H3 in OC cells. (A) Schematic illustration of human *Bcl3* promoter showing potential STAT1 and NFκB binding sites. (B) ChIP analysis of human *Bcl3* promoter occupancy by STAT1, Ser727 pSTAT1, p65, K314/315 ac‐p65, histone H3, and K9 ac‐histone H3 in IFNγ (50 ng·mL^−1^)‐treated SKOV3 cells. The data are presented as fold difference in occupancy of the particular protein at the *Bcl3* promoter compared with the negative control human IGX1A (SA Biosciences) locus, which does not contain any transcription factors binding sites. The values represent the mean ± SE (*n* = 3); one‐way ANOVA Tukey *post hoc* test; an asterisk denotes a statistically significant (*P < 0.05) change compared with UT cells (*T* = 0). (C) Western analysis of STAT1, Ser727 pSTAT1, p65, K314/315 ac‐p65, histone H3, K9 ac‐histone H3, and control Actin in WCE of SKOV3 cells incubated with 50 ng·mL^−1^ IFNγ.

In most cell types, IFNγ induces gene expression by STAT1 promoter binding, and in many cases, STAT1 phosphorylation at Ser727 is required for its full transcriptional and biological activity [29]. Using MEME Suite and JASPAR databases, we have identified a potential STAT1 binding site in human *Bcl3* promoter, located ‐418 nucleotides from the transcription start site (TSS) **(**Fig. 5A **)**. Thus, using ChIP, we analyzed whether IFNγ increases STAT1 and Ser727 pSTAT1 (pSTAT1) occupancy at this site. In addition, since the IFNγ‐induced Bcl3 expression was dependent on p65 NFκB (Fig. 4), we analyzed whether IFNγ induces a direct recruitment of p65 or its transcriptionally active K314/315 acetylated form (ac‐p65) to the previously identified p65 NFκB binding site located ‐289 nucleotides from TSS [8]. As shown in Fig. 5B, IFNγ increased recruitment of pSTAT1, but not unphosphorylated STAT1 to *Bcl3* promoter, while neither p65 nor ac‐p65 were recruited. However, IFNγ increased *Bcl3* promoter occupancy by Lys9 acetylated histone H3 (ac‐H3) (Fig. 5B), consistent with active transcription.

The increased promoter occupancy by pSTAT1 and ac‐H3 (Fig. 5B) suggested that IFNγ might increase STAT1 phosphorylation and histone H3 acetylation; alternatively, IFNγ could just promote *Bcl3* promoter occupancy by pSTAT1 and ac‐H3, without increasing their total cellular levels. To distinguish between these two possibilities, we analyzed STAT1, p65, and histone H3 total cellular levels by western blotting. As shown in Fig. 5C, IFNγ increased total cellular levels of pSTAT1 as well as unphosphorylated STAT1. In addition, as previously observed [40], IFNγ increased the protein expression of p65, but not its K314/315 acetylation. Neither total histone H3 protein levels nor its Lys9 acetylation were affected by IFNγ treatment in OC cells (Fig. 5C). Together, these data indicate that IFNγ induces the Bcl3 expression in OC cells by promoting the STAT1 expression and its Ser727 phosphorylation and recruitment to *Bcl3* promoter, which is facilitated by a concomitant IFNγ‐induced promoter acetylation.

### Bcl3 promotes expression of IL‐8/CXL8 in OC cells

**Fig. 6:**
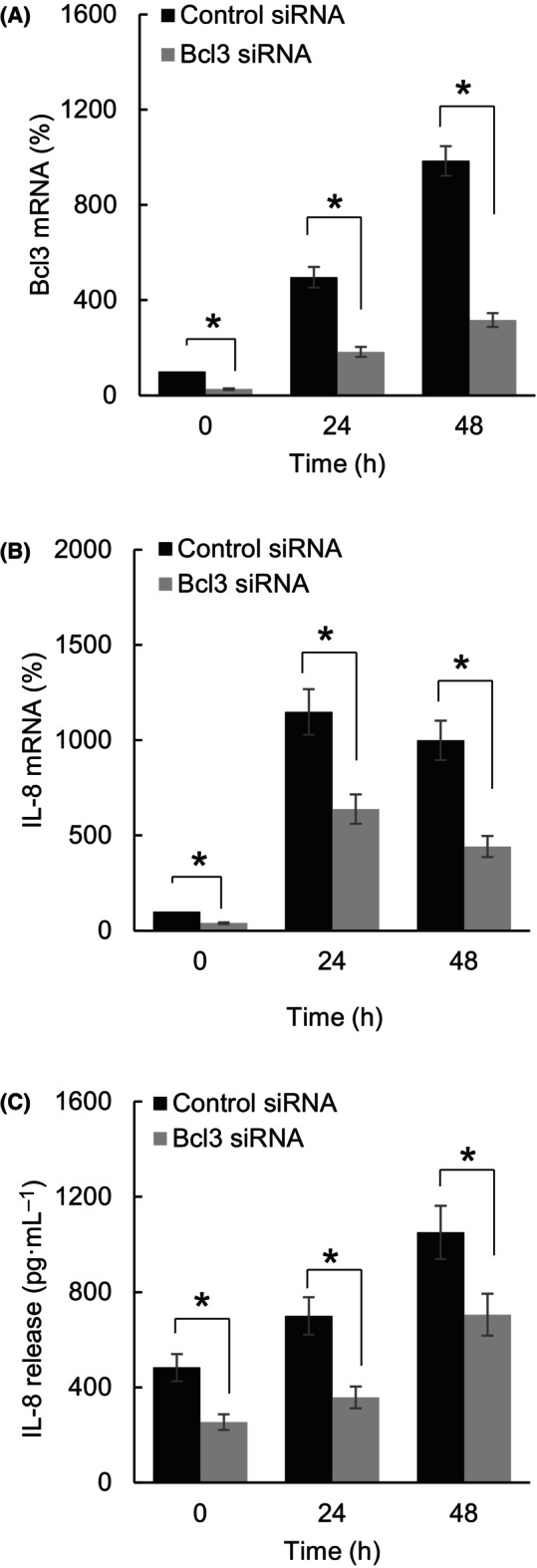
Bcl3 promotes expression of IL‐8/CXL8 in OC cells. RT‐PCR of Bcl3 (A) and IL‐8 (B) mRNA levels in SKOV3 transfected with control or Bcl3 siRNA and treated with IFNγ (50 ng·mL^−1^). (C) ELISA of IL‐8 cytokine release in SKOV3 transfected with control or Bcl3 siRNA and treated with IFNγ (50 ng·mL^−1^). The values represent the mean ± SE (*n* = 3); unpaired *t*‐test; an asterisk denotes a statistically significant (*P < 0.05) change compared with cells transfected with control siRNA.

We have previously shown that IFNγ induces PD‐L1 expression that is dependent on Bcl3 in OC cells [9]. In addition, we have recently found that IFNγ induces expression of the pro‐inflammatory and pro‐angiogenic chemokine IL‐8/CXCL8, resulting in increased migration and invasion of OC cells [40]; however, the mechanisms that regulate the IFNγ‐induced IL‐8 expression remain largely unknown. Since the levels of PD‐L1 and IL‐8 correlate in cancer patients [44, 45, 46], we hypothesized that the IFNγ‐induced IL‐8 expression in OC cells might be also mediated by Bcl3. Indeed, Bcl3 suppression by siRNA significantly decreased both constitutive and IFNγ‐induced IL‐8 mRNA expression and cytokine release in OC cells (Fig. 6), indicating that Bcl3 promotes the IL‐8 expression.

## Discussion

Increased Bcl3 expression in cancer cells promotes their proliferation, migration, and metastatic potential, but the mechanisms that regulate the Bcl3 expression in solid tumors are incompletely understood. We have recently shown that the Bcl3 expression is induced by IFNγ in OC cells, resulting in their increased proliferation and PD‐L1 expression [9]. In this study, we show that the IFNγ‐induced Bcl3 expression is dependent on JAK1/STAT1 and p65 NFκB, and promotes expression of the pro‐inflammatory chemokine IL‐8. To our knowledge, this is the first study that identifies Bcl3 as a target of the JAK1/STAT1 signaling, and inducer of the IL‐8 expression.

Our data indicate that IFNγ induces the Bcl3 expression by increasing acetylation of the *Bcl3* promoter, with a simultaneous promoter recruitment of Ser727 pSTAT1. However, even though the IFNγ‐induced Bcl3 expression in OC cells is also dependent on p65 NFκB, we did not observe p65/ac‐p65 recruitment to the ‐289 p65 NFκB binding site in *Bcl3* promoter. It is possible that p65 NFκB might mediate the IFNγ induced Bcl3 expression through its binding to other NFκB binding site(s) in *Bcl3* promoter, or to a downstream enhancer. This hypothesis is supported by a previous study that showed that NFκB induces *Bcl3* transcription through an intronic enhancer [10]. Alternatively, p65 NFκB might facilitate *Bcl3* transcription through a cooperative interaction with STAT1. Indeed, several studies have demonstrated a crosstalk between NFκB and STAT1 signaling in the regulation of IFNγ‐induced inflammatory genes [30, 47, 48, 49].

Interestingly, in the immunosuppressive cutaneous T‐cell lymphoma cells, Bcl3 inhibits expression and release of the pro‐inflammatory chemokine IL‐8 [8]. However, our present findings indicate that Bcl3 induces the IL‐8 expression in OC cells. The IL‐8 expression is increased in OC and other solid tumors, where it induces cancer progression through its induction of tumor cell proliferation, migration, invasion, and immune escape [50, 51, 52, 53, 54]. Increased IL‐8 serum levels reflect tumor burden and correlate with poor prognosis in OC and other solid tumors [44, 45, 54]. Since both Bcl3 and IL‐8 are IFNγ‐inducible genes that promote tumor cell proliferation, migration, and invasion [9, 40], the positive regulation of IL‐8 by Bcl3 suggests that the Bcl3 oncogenic function in OC cells might be partly mediated by its upregulation of IL‐8.

Because of its anticancer properties, IFNγ has been used in cancer treatment [32, 33, 34, 35]. In addition, IFNγ expression is induced by radiation and ICB in cancer treatment [36, 37, 38]. However, IFNγ also has important tumor‐promoting functions that include the induced PD‐L1 and IL‐8 expression resulting in increased cancer cell proliferation and immune escape, but the mechanisms are much less understood [9, 40]. Understanding the tumor‐promoting mechanisms and molecular targets of IFNγ is crucial to minimize its tumor‐promoting functions in IFNγ‐based therapies, and in cancer treatments associated with IFNγ increase.

Our present findings are the first to demonstrate that the IFNγ‐induced Bcl3 expression is dependent of JAK1/STAT1 and p65 NFκB signaling. In addition, our *in vitro* data show that Bcl3 promotes the IL‐8 expression in OC cells. Together, these results suggest that targeting the IFNγ‐induced JAK1/STAT1/p65 NFκB signaling may suppress the IFNγ‐induced Bcl3 and IL‐8 expression in OC. Future studies should extend these *in vitro* findings and determine whether IFNγ induces the Bcl3 and IL‐8 expression in animal models and clinical samples.

## Conflict of interest

The authors declare no conflict of interest.

### Peer review

The peer review history for this article is available at https://www.webofscience.com/api/gateway/wos/peer‐review/10.1002/2211‐5463.13624.

## Author contributions

BG and IV designed the experiments. BG, SP, YZ, MMU, and SUR performed experiments. BG and IV analyzed the data and wrote the manuscript. IV obtained funding and supervised the project. All authors approved the final version of the manuscript.

## Data accessibility section

The data that support the findings of this study are available from the corresponding author upon reasonable request.
